# MMS observations of electron scale magnetic cavity embedded in proton scale magnetic cavity

**DOI:** 10.1038/s41467-019-08971-y

**Published:** 2019-03-04

**Authors:** H. Liu, Q.-G. Zong, H. Zhang, C. J. Xiao, Q. Q. Shi, S. T. Yao, J. S. He, X.-Z. Zhou, C. Pollock, W. J. Sun, G. Le, J. L. Burch, R. Rankin

**Affiliations:** 1 Institute of Space Physics and Applied Technology, Peking University, Beijing, 100871 China; 2 Geophysical Institute, University of Alaska Fairbanks, Fairbanks, AK 99775 USA; 3 State Key Laboratory of Nuclear Physics and Technology, School of Physics, Peking University, Beijing, 100871 China; 4 Shandong Provincial Key Laboratory of Optical Astronomy and Solar-Terrestrial Environment, Institute of Space Sciences, Shandong University, Weihai, 264209 China; 5 Denali Scientific, 3771 Mariposa Lane, Fairbanks, AK 99709 USA; 6 Department of Climate and Space Sciences and Engineering, University of Michigan, Ann Arbor, MI 48109 USA; 7 NASA, Goddard Space Flight Center, Greenbelt, MD 20771 USA; 8 Southwest Research Institute, San Antonio, TX 78238 USA; 9 Department of Physics, University of Alberta, Edmonton, T6G 2G7 AB Canada

## Abstract

Magnetic cavities (sometimes referred to as magnetic holes) at electron kinetic scale are thought to be one of the extremely small intermittent structures formed in magnetized turbulent plasmas, where the turbulence energy cascaded down to electron scale may finally be dissipated and consequently energize the electrons. However, the geometry and formation of these structures remain not definitively resolved. Here we discuss an electron scale magnetic cavity embedded in a proton scale magnetic cavity observed by the MMS spacecraft in the magnetosheath. By applying an innovative particle sounding technique, we directly depict the boundary of the electron scale magnetic cavity and uncover the geometry. We find that this structure is nearly circular with a radius of 10.0 km and its formation is due to the diamagnetic current. Investigation of the electron scale structure is only recently made possible by the high spatial and temporal resolution provided by MMS observations.

## Abstract (web summary)

Magnetic cavities are universal phenomena existing in cosmic plasma environments. Here Liu et al. show electron scale magnetic cavities in proton scale magnetic cavities observed by Magnetospheric Multiscale (MMS) spacecraft in the Earth’s magnetosheath, and depict the boundary of the electron scale magnetic cavity using particle sounding technique.

## Introduction

Sudden dips of the magnetic field strength have been broadly discussed in solar-terrestrial space plasmas. A descriptive phrase magnetic hole was first proposed to describe these dips in the solar wind^1^, while the mechanisms have not been fully settled yet. It was recently recommended that magnetic dips in the interplanetary space but not mirror mode waves should be referred as magnetic decreases, for which a variety of mechanisms could serve as the explanations^2^. The mirror modes^3^ may also create dips in the magnetic field strength, which have been commonly identified in planetary magnetosheaths (e.g. in the Earth^2^, Jupiter^4,5^, Saturn^4,6,7^) and in the heliosheath^2,8^. The scale of these structures varies from several to thousands of proton gyro-radii^9^. It is also suggested that mirror structures are one of the coherent structures caused by nonlinear energy cascade in magnetized turbulent plasmas^10,11^. In contrast, a simulation showed that mirror modes would expand due to a Bohm-like diffusion process, during which the structures could maintain their characteristics and not become turbulent^12^.

In recent years, it has attracted a lot of interest in understanding energy cascade in turbulent plasma from magnetohydrodynamic scales to kinetic scales. Observations in solar wind and terrestrial magnetosheath by Cluster^13^, Wind^14,15^, THEMIS^16^, and MMS^17,18^ have been reported. Meanwhile, evidence for coherent structures from ion scales to electron scales has been shown^19^, and the turbulent energy is thought to be finally dissipated at electron scales^20^. However, scales of energy cascade and energy dissipation mechanism in turbulent plasma are still outstanding questions.

Recently, observations and simulations of small-scale magnetic cavities, sudden dips at scales smaller than proton thermal gyro-radii which are commonly referred to as small-scale magnetic holes, were reported. A case study of a sub-proton scale structure was first presented in 2011 with observations in the plasma sheet^21^, followed by a statistical study^22^. A particle-in-cell simulation^23^ showed the structures formed in decaying turbulent plasma, and the formation and stability were due to a vortex of trapped electrons, which locally enhance the temperature and pressure perpendicular to the magnetic field. The contribution to intermittency of turbulence was also suggested^23^. Similar structures were reported later in three-dimensional (3D) simulations of turbulence^24^, but without any detailed explanation for the mechanism. Later, these structures were studied using a multi-spacecraft mapping method^25^, showing that at least some events were consistent with the model of decaying turbulence^23^. Then a series of papers followed with observations in the terrestrial magnetosheath^26–28^ and the plasma sheet^29–32^, which support the model of turbulence^23^, meanwhile an assumption of circular cross-section was often made. However, there are still a lot of outstanding questions about magnetic cavities, for example, the generation mechanisms, of which the candidates include mirror mode instability^21,22^ and the magnetosonic soliton model^33^, while both mechanisms have been questioned in the literature^27,34^. Other outstanding questions include the morphology, and the distributions and evolutions of the plasmas within these structures.

The Magnetospheric Multiscale (MMS) spacecraft, launched on 12 March 2015, provide plasma data with high spatial and temporal resolution, enabling multi-point observations of very small-scale structures^35^. The Fast Plasma Investigation (FPI) instrument reports a measurement of a full 3D electron phase space density (PSD) distribution every 30 ms in its burst data product, at least 100 times faster than previous missions^36^. It also contains a high angular resolution, 32 × 16. The MMS mission has significantly promoted study of small-scale magnetic structures.

The energetic particle sounding technique (or sounding for short) was first proposed and successfully applied to the magnetopause^37,38^. As illustrated in the Methods section, this technique can be used to determine the boundary orientation and distance to the spacecraft based on observed non-gyrotropic distributions of energetic particles which are caused by a sharp boundary (called finite Larmor radius effect) close to the spacecraft (within twice of the gyro-radius). It has been applied to remotely sound the terrestrial magnetopause^39,40^. However, applications of the sounding technique have been limited to highly energetic particles (above the FPI energy range) and large-scale structures by insufficient time and angular resolution, especially for electron data, until the MMS mission. MMS data are perfect for employing this sounding technique, especially to small-scale structures, using the high-resolution plasma measurements from FPI.

In this paper, we determine the geometry of an electron scale magnetic cavity (ESMC) using the sounding technique, showing that the boundary is nearly circular with a radius of 10.0 km. We then obtain the distributions of plasma parameters inside the ESMC and discuss the generation mechanism and evolution of the ESMC.

## Results

### Overview of the structures at proton and electron scales

The MMS spacecraft follow an elliptic orbit around the Earth. They were in the dayside magnetosheath at around 1500 Universal Time (UT) on 23 October 2015. The time interval discussed in this paper extends from 14:59:20 to 14:59:50 UT, when the magnetic local time is 14.9, and the magnetic latitude is −23.5° (i.e. in the Southern Hemisphere). In this paper, we use burst mode electron distribution function data (30 ms time resolution) as well as electron moments from the FPI^36^, and burst type magnetic field data from the fluxgate magnetometers (FGM)^41^ (7.8 ms time resolution) and search coil magnetometer (SCM)^42^ (0.12 ms time resolution). All data used for this study are publicly available to download from the MMS Science Data Center (https://lasp.colorado.edu/mms/sdc/public/). This event has been discussed previously^26^ as a first report of a small-scale magnetic cavity in the magnetosheath, which is characterized by a dip in the magnetic field strength, enhanced electron flux at 90°, and the electron vortex.

**Fig. 1:**
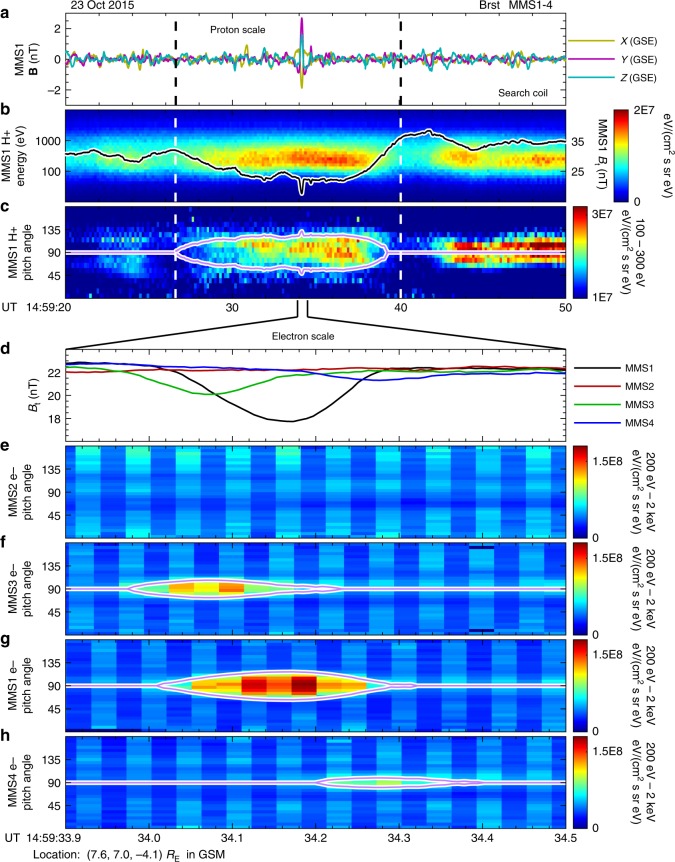
MMS observations of an ESMC coupled with a proton scale magnetic cavity. Panels **a**–**c** show MMS1 observations of turbulent magnetic fields, proton energy–time spectrogram as well as the total magnetic field (*B*_t_), and proton pitch angle distribution (PAD) in 30 s from 14:59:20UT to 14:59:50 UT on 23 October 2015. A depression region of *B*_t_ is marked with two dashed vertical lines across the four panels. The *B*_t_ observed by MMS1 is over-plotted as a black curve in panel **b**. The magenta curves in panel **c** represent a local loss cone, derived from the variation of *B*_t_. The ESMC discussed in this paper is the first one of two sudden dips of *B*_t_ near 14:59:34 UT in panel **b**, the observations of which are detailed in panels **d**–**h**, with a very short time scale (0.6 s). Panel **d** shows *B*_t_ observed by four MMS spacecraft, and panels **e**–**h** show electron PADs observed by MMS2, 3, 1, and 4, respectively. In panels **f**–**h** the corresponding local loss cones are over-plotted as magenta curves. Spacecraft positions are labeled at the bottom of the figure

We start with introducing observational characteristics of the proton scale structure and then zoom into the electron scale phenomenon. Figure 1 shows MMS observations of the magnetic field, the proton energy spectrogram, and the pitch angle distributions (PADs) of protons and electrons as functions of UT. The time scale is 30 s in panels a–c. Panels a and b show that there is a region of magnetic field strength (*B*_t_) depression between dashed lines together with an energy flux enhancement of the protons between 100 to 1000 eV in the energy–time spectrogram. In fact, the *B*_t_ depression is balanced by the intensive proton thermal pressure (shown in panel b of Supplementary Fig. 1). It is further illustrated by the proton PAD in panel c that the enhancement of proton energy flux occurs mainly in the direction perpendicular to the magnetic field. The magenta curves in panel c represent a local loss cone, which is derived from the observed magnetic fields:

1 $${\mathrm{sin}}(\alpha _{\mathrm{l}}) = \sqrt {\frac{{B_{\mathrm{t}}}}{{B_{\mathrm{o}}}}},$$

where *B*_t_ is the observed magnetic field strength shown in panel b, and *B*_o_ (31.8 nT) is a constant ambient magnetic field strength outside the depression region. The region enclosed by magenta curves in panel c is an expected trapping region by the magnetic mirror force, which agrees well with observations of the proton energy flux enhancement. The simultaneous magnetic field depression and enhancement in the proton energy flux indicate the existence of a proton scale magnetic cavity (PSMC) between the two dashed lines.

We found that the PSMC is coupled with a much smaller (electron scale) magnetic structure. There are two prominent sudden dips of *B*_t_ near 14:59:34 UT in panel b. It is illustrated below that the first dip is also a magnetic cavity, which is embedded inside the proton scale structure. Panels d–h show observations of the electron scale structure during a very short time scale (0.6 s). The *B*_t_ of MMS2 remains unchanged during this period, but depressions for MMS3, 1, and 4 are clear (panel d). In the meantime, there are significant enhancements of PADs for MMS3, 1, and 4 as illustrated in panels f–h, while the PAD for MMS2 shows no obvious disturbance (panel e). Similar to panel c, the corresponding local loss cones are over-plotted in panels f–h, exactly enclosing regions of enhanced PADs. The magnetic field directions observed by all spacecraft change little (less than 5°, shown in panel d of Supplementary Fig. 1) during this time period, thus this event could be referred as a linear event^2,43^. The combined observations of *B*_t_ depressions, unchanged magnetic directions, and simultaneous PAD enhancements indicate that the very small-scale structure is a linear magnetic cavity, referred to as ESMC in this paper. The absence of *B*_t_ depression and PAD enhancement suggests that the MMS2 spacecraft did not encounter the ESMC.

Before further discussion, we would like to define a plasma rest frame for convenience. Since the proton bulk velocities (*V*_p_) for all MMS spacecraft are nearly constant (while electron bulk velocities are not), we let the frame move with protons. We first define the *Z*-axis by the average direction of the magnetic field from all four MMS spacecraft between 14:59:33.9 and 14:59:34.5 UT. Then we let the *X*-axis to be anti-parallel to a component of the average *V*_p_ that is perpendicular to the average magnetic field. Finally, the *Y*-axis completes the orthogonal set. All observations and calculations presented in the remainder of this paper are re-organized into this plasma rest frame.

To determine the geometry of a magnetic cavity, there are some traditional approaches such as mapping techniques^25,29^. In this paper, we use a completely different method, the particle sounding technique, to directly determine the geometry without such an assumption. Since this technique relies on non-gyrotropy of particle distributions, we present the 3D PSDs in the next section.

### MMS observations of non-gyrotropy of electron PSDs

**Fig. 2:**
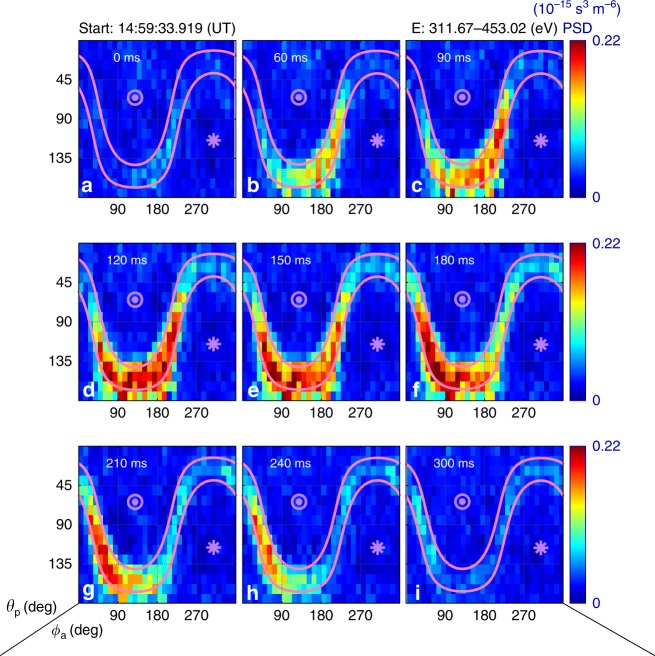
Sky-maps of electron PSD from MMS3 for one energy channel. Panels **a**–**i** show observations at different times. For each panel the horizontal and vertical axes are azimuthal (*ϕ*_a_, degrees) and polar angles (*θ*_p_, degrees), respectively. The measuring time is labeled at the top of each panel, starting from 14:59:33.919 UT. Magenta lines represent the local loss cone. The asterisk and the circle mark directions parallel and anti-parallel to the magnetic field, respectively. The energy channel is labeled in the top right corner of the figure. All panels share the same color bar

In Fig. 2, electron PSDs from MMS3 are organized as sky-maps for electrons with energies between 312 and 453 eV. Panels a–i show observations at different times. In each panel, two magenta lines represent the local loss cone, between which it is a trapping region where electrons are confined by the local magnetic mirror force. At the beginning (panel a) when the spacecraft was outside the ESMC, there was no obvious enhancement throughout the map. A clear enhancement of PSD in the trapping region is visible in panel b when the spacecraft entered the structure. The non-gyrotropy of the enhanced PSD is significant, as we expected. Then the enhanced region deepens and slightly expands with time, always exhibiting non-gyrotropic signature, and are confined in the trapping region. Finally, the enhancement disappears in panel i when the spacecraft exits the ESMC.

Data from only one energy channel are shown in Fig. 2 as an example. The non-gyrotropic distributions are observed across several energy channels. For PSDs observed by MMS1 and MMS4, there are similar phenomena simultaneously with the *B*_t_ dips. Note that there is nothing for MMS2, as expected. Sky-maps for all spacecraft and several energy channels could be found in Supplementary Figs. 2–4.

### Geometry of the ESMC derived from the sounding technique

**Fig. 3:**
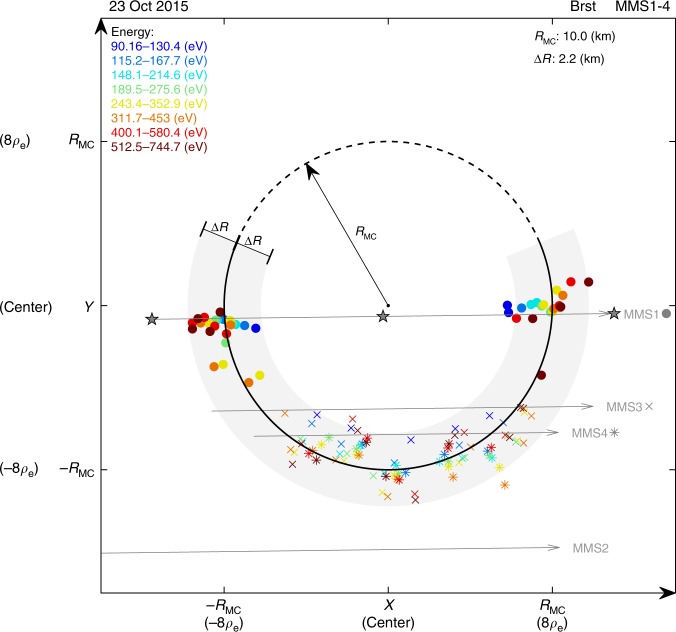
Geometry of the electron scale magnetic cavity obtained by sounding technique. All data are re-organized in *XY* plane of the plasma rest frame. Solid circles, crosses, and asterisks represent MMS1, MMS3, and MMS4, respectively. Different colors denote different energy channels, labeled in the top left corner. A circle (*R*_MC_, ∼8*ρ*_e_) that best fits the data points was obtained. The solid part of the circumference is surrounded by data, where a light gray region illustrates the uncertainty (∆*R*). The radius and the uncertainty are labeled in the top right corner. Spacecraft trajectories are plotted as gray arrowed lines. *ρ*_e_ is the gyro-radius of a thermal electron in the event discussed in this paper

As mentioned above, the electron non-gyrotropy can be utilized to remotely sound the ESMC boundary. A clear geometry of the structure has been obtained by the sounding technique. For the ESMC event discussed in this paper, this technique generates more than one hundred data points in the plane perpendicular to the magnetic field, i.e., the *XY* plane of the plasma rest frame. They are plotted in Fig. 3.

In Fig. 3, data points from four spacecraft and eight energy channels are differentiated by different symbols and colors, detailed in the caption. It is surprising that all data points form a semicircle. A circle that best fits the data points is obtained. The center of the circle is regarded as the center of the ESMC. The radius of the circle (*R*_MC_) is 10.0 km, which is approximately eight times the gyro-radius of a thermal electron (*ρ*_e_ ~1.25 km). The dashed arc represents the segment where observations were not available. The gray region surrounding the solid arc represents an uncertainty (∆*R* ∼2.2 km) estimated from Eq. (7), covering 95.9% of the data points.

It is also worth mentioning that the boundary is assumed to be a planar for the application of the sounding technique, while the final result shows that it is a circle. Since the boundary shape is unknown before we get the result, assuming a planar boundary should be the best choice, but it may underestimate the distance for an actual boundary curved outward. Thus, a correction method is proposed (Eqs. (8)–(10)) in the Methods section), which has already been applied to the data points shown in Fig. 3. The underestimation is evaluated to be about 0.47 km based on Eqs. (9) and (10), which is not significant compared with ∆*R*, the uncertainty of the sounding result.

The spacecraft trajectories are over-plotted as gray arrowed lines in Fig. 3. It is shown that the trajectory of MMS2 has no intersection with the ESMC, consistent with the absence of any signature in MMS2 observations. Since the radius of the structure is smaller than the gyro-radius of a 4 keV electron, we prefer to use the term electron scale rather than small scale, sub-proton scale, or kinetic-size.

### The energy dependence of the enhancement of PSD_⊥_

**Fig. 4:**
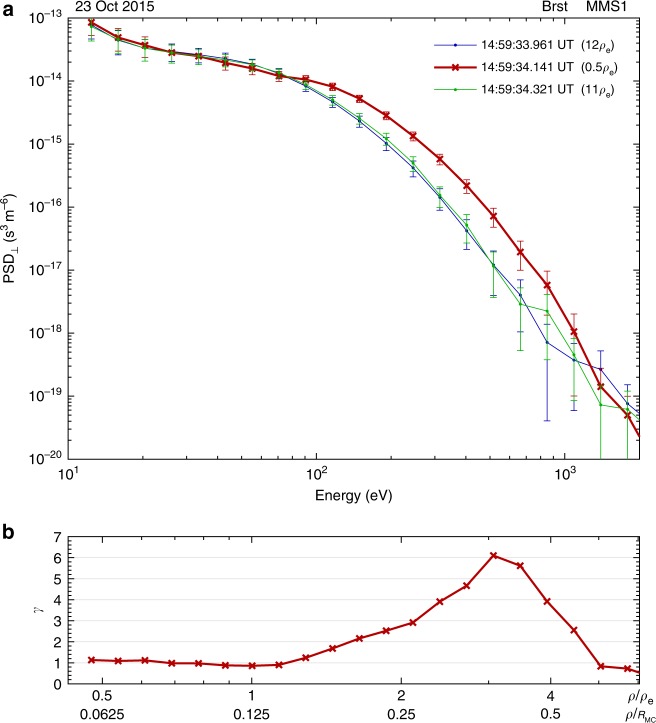
The energy spectra of electron PSD_⊥_ observed inside and outside the ESMC. PSD_⊥_ is averaged over 360° in the plane perpendicular to the magnetic field. In panel **a**, the *x*-axis is the electron energy, and the *y*-axis is the average PSD_⊥_ (with error bars). The red line represents observations taken near the center of the ESMC, and the other two lines (blue and green) represent observations outside the structure. The time of measurement and the distance between the spacecraft and the center of ESMC are labeled in the top right corner. In panel **b**, the *x*-axis is the same as that in panel **a**, but the energy is reformulated in terms of electron gyro-radius *ρ* and normalized by the gyro-radius of the thermal electron (*ρ*_e_) or the radius of the magnetic cavity (*R*_MC_). *γ* is the ratio between the PSD_⊥_ inside (red line in panel **a**) and outside (blue and green lines in panel **a**) the structure in each energy channel. The error bars in panel **a** are instrumental errors directly obtained from the FPI data product

In panel a of Fig. 4, the red line is the energy spectrum of the average PSD_⊥_ (in the plane perpendicular to the magnetic field) closest to the center of the ESMC, and the blue and the green lines represent observations outside the ESMC. All the three lines are from MMS1 observations, and the corresponding spacecraft locations are labeled as pentagrams along MMS1 trajectory in Fig. 3. In panel b, it is demonstrated by a parameter *γ*, defined as the ratio between the PSD_⊥_ inside and outside the ESMC, that the enhancement of PSD_⊥_ occurs from 0.1 to ∼0.7 *R*_MC_.

## Discussion

**Fig. 5:**
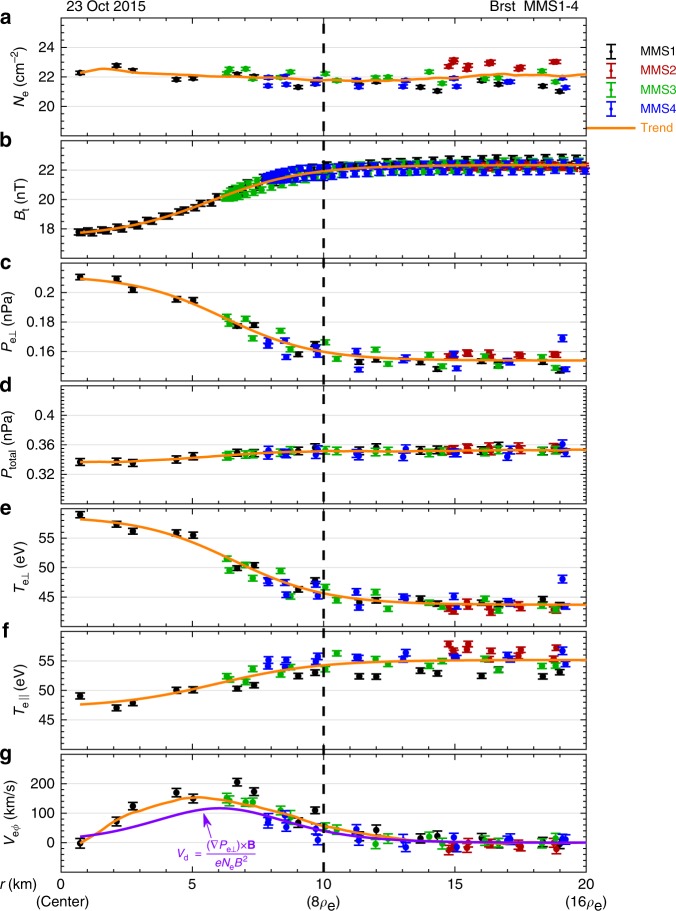
Radial distributions of the electron plasma parameters and the magnetic fields. Panels **a**–**g** show electron number density (*N*_e_), magnetic field strength (*B*_t_), perpendicular pressure (*P*_e⊥_), total pressure (*P*_total_), perpendicular temperature (*T*_e⊥_), parallel temperature (*T*_e||_), and electron tangential bulk velocity (*V*_eϕ_). The *x*-axis is the distance (*r*) between the measuring position and the center of the ESMC. In each panel, solid dots with error bars represent direct observations, and the spacecraft are labeled as different colors. The orange curves represent a trend of the collective. In panel **g**, the purple curve is a diamagnetic drift velocity (*V*_d_) estimated from *P*_e⊥_. The dashed line marks the radius of the ESMC (i.e., *R*_MC_) obtained by the sounding technique. The error bars are instrumental errors directly obtained from the FPI data product except for *P*_total_, which is derived from its definition and the propagation of error

In this paper, we not only directly determine the geometry of the ESMC, but also precisely obtain the spacecraft positions relative to the structure (Fig. 3). It is also demonstrated that the ESMC discussed in this paper is quasi-static and frozen-in to protons in the time scale of 0.6 s, with a circular boundary. Figure 5 shows spatial distributions of some key plasma parameters related to the ESMC.

In Fig. 5, the *x*-axis (*r*) is the distance between the spacecraft and the center of the ESMC. That is, *r* *=* 0 is the center of the ESMC, and *r* *=* 10.0 km represents the boundary. In panels a–g, parameters plotted as functions of *r* include: electron number density (*N*_e_), magnetic field strength (*B*_t_), perpendicular pressure (*P*_e⊥_), total pressure (*P*_total_ *=* *B*^2^*/*(2*µ*_0_) + *P*_e⊥_), perpendicular temperature (*T*_e⊥_), parallel temperature (*T*_e||_), and electron tangential bulk velocity (*V*_eϕ_). It is shown that some parameters either increase or decrease with *r*, such as *B*_t_, *P*_e⊥_, *T*_e⊥_, and *T*_e||_, but *N*_e_ and *P*_total_ remain nearly unchanged. *V*_eϕ_ increases with *r* at distances less than 5 km and decreases with *r* at distances larger than 5 km. In each panel, the orange curve going through the data points can well represent the trend. For *N*_e_, *P*_total_, and *V*_eϕ_ the orange curves are smooth of the data, while for *B*_t_, *P*_e⊥_, *T*_e⊥_, and *T*_e||_ they are derived by fitting observational data to tanh functions, since it has been shown that magnetic decreases could be modeled as current sheets^43^.

In panel g of Fig. 5, the purple curve represents a diamagnetic drift velocity (*V*_d_) estimated from *B*_t_ and *P*_e⊥_. It is derived from the momentum equation of a certain species:

2 $$N_{\mathrm{s}}\frac{{{\mathrm d}{\mathbf{v}}_{{{\mathrm s}}}}}{{{\mathrm {d}}t}} = - \frac{1}{{m_{\mathrm{s}}}}\nabla \cdot {\mathbf{P}}_{\mathrm {s}} + \frac{{q_{\mathrm{s}}N_{\mathrm{s}}}}{{m_{\mathrm{s}}}}\left( {{\mathbf{E}} + {\mathbf{v}}_{\mathrm{s}} \times {\mathbf{B}}} \right),$$

where **v**_s_ is the bulk velocity, *N*_s_ is the number density, **P**_s_ is the pressure tensor, and **E** is the electric field. Assuming the structure is quasi-static (i.e., d**v**_s_/d*t* *=* 0) and ignoring the electric drift term, the perpendicular bulk velocity (*v*_s⊥_) should be the same as the diamagnetic drift velocity (*V*_d_):

3 $$v_{{\mathrm{s}} \bot } = V_{\mathrm{d}} = - \frac{{\left| {\left( {\nabla \cdot {\mathbf{P}}_{\mathrm{s}}} \right) \times {\mathbf{B}}} \right|}}{{q_{\mathrm{s}}N_{\mathrm{s}}B_{\mathrm{t}}^2}}.$$

It is shown in panel g of Fig. 5 that the estimated *V*_d_ roughly matches the observed tangential bulk velocity *V*_eϕ_, confirming that the toroidal current carried by electrons are generated from diamagnetic effect. A slight underestimation of *V*_eϕ_ suggests that the ESMC discussed in this paper is slowly shrinking, but on a time scale much longer than observations of the ESMC (0.6 s).

If we take the Ampère circuital theorem into account, considering that the protons would make no contribution to the total current in the plasma rest frame, an equilibrium equation should be:

4 $$\frac{\partial }{{\partial r}}\left( {P_{{\mathrm{e}} \bot } + \frac{{B_{\mathrm{t}}^2}}{{2\mu _0}}} \right) = \frac{{\partial P_{{\mathrm{total}}}}}{{\partial r}} = 0.$$

It agrees well with the observation that the *P*_total_ changes little with *r* (Fig. 5d). Thus, it is confirmed that the ESMC is quasi-static and the electron vortex seems to maintain its structure self-consistently. Similarly, it is shown in panel b of Supplementary Fig. 1 that the proton scale magnetic field depression is balanced by proton pressure.

**Fig. 6:**
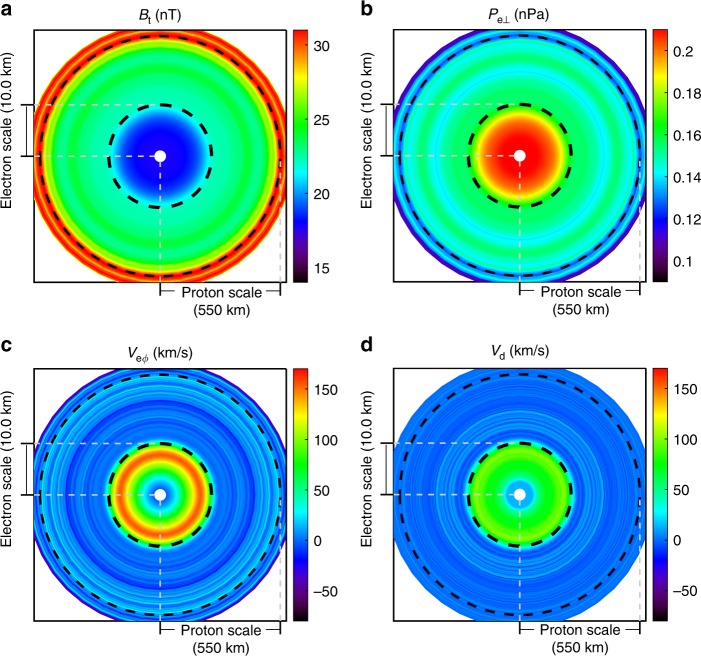
The electron scale magnetic cavity embedded in the proton scale magnetic cavity. Panels **a**–**d** denote the magnetic field strength (*B*_t_), electron perpendicular pressure (*P*_e⊥_), tangential bulk velocity (*V*_eϕ_), and the estimated diamagnetic drift velocity (*V*_d_), respectively. In each panel, the origin is set to be the center of the ESMC, and the distance is in log scale (from 0.5 to 780 km). Each 2D distribution, derived from observations, is regarded as a function of the distance to the center of the ESMC. The small white region near the center is due to the absence of observations

In this paper, we report an ESMC embedded in a PSMC, though the generation mechanisms of both are not fully understood. It has been shown in Fig. 1 that the PSMC is characterized by magnetic depression and trapped protons in the local mirror. Although the proton data here are not suitable for the sounding technique (due to a field-aligned bulk velocity of the same order as the proton thermal velocity), we can estimate the scale assuming it is moving along with the proton plasma in the magnetic perpendicular plane that $L\sim \Delta t\cdot V_{{\mathrm{p}} \bot }/2 \approx 550\;{\mathrm {km}} \approx 5\cdot \rho _{\mathrm{p}}$, where *V*_p⊥_ is the perpendicular proton bulk velocity and *ρ*_p_ is the proton thermal gyro-radius (*ρ*_p_ ~ 112 km). The whole picture is schematically plotted in Fig. 6 on log scales. For the ESMC event the scale size is between electron (~1.25 km) and ion (~112 km) thermal gyro-radius, substantially smaller than ion inertial length of 48.5 km (but larger than electron inertial length of ~1.13 km), thus the term electron scale should be appropriate.

The generations of these macroscale or microscale magnetic structures are complex problems. There are many proposed generation mechanisms^2^ for macroscale magnetic decreases, such as nonlinear Alfven waves^44,45^, mirror instability^46^, the magnetosonic soliton model^33^, large-amplitude Alfven wave packets^34^, wave–wave interactions behind interplanetary shocks^47^, etc. Our observations of the nested morphology suggest the coupling of macroscale and microscale phenomena. For the ESMC event discussed in this paper, it seems that the propagating solitons can be excluded since the structure is quasi-static and non-propagating in the plasma rest frame. The electron mirror instability is not an authorized explanation either, in case of the parallel electron temperature exceeding the perpendicular temperature in the background.

The electron scale magnetic cavities have been successfully generated in simulated decaying plasma compressible turbulence in a 3D fully kinetic framework^24^. The observations in this paper strongly confirm most of their simulation results, such as the electron vortex, the total pressure balance, the quasi-static equilibrium, and the partly enhanced PSD inside the structure. It is also shown in simulations that the structure has a circular cross-section with a scale size similar to the gyro-radius of trapping electrons^23^, consistent with our observations. However, the electron PSD in simulations did not show a clear local loss cone structure which is evident in observations presented in this paper, indicating potential improvements of simulations, such as a longer simulating period.

We directly determine the geometry of the ESMC and the distributions of plasma parameters using the sounding technique based on high-resolution MMS data, confirming the significance of compressible turbulence (existence of small-scale divergence of the pressure tensor) in generating such long-lasting electron scale magnetic cavities. The spatial distributions of electron plasma moments are expressed as functions of radial distance in the structure rest frame in Fig. 5, showing the symmetry and the quasi-steady state of the structure, the pressure balance, and the electron current due to diamagnetism. The geometry and the dynamics uncovered confirms the model of decaying turbulence^23,24^ forming the ESMCs as coherent structures in turbulent plasma. Furthermore, the suggestion of the coupling of different scale phenomena might be a way of cross-scale energy transport in turbulent cascade. A possible explanation is that the proton scale cavity can provide a high electron plasma beta environment, facilitating the generation of diamagnetic currents in electron scale.

## Methods

### Principle of the energetic particle sounding technique

**Fig. 7:**
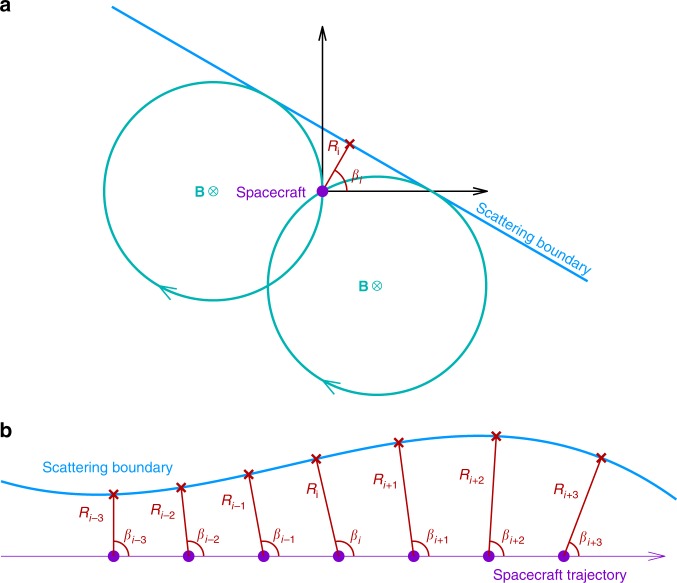
Schematic diagram of the energetic particle sounding technique. In panel **a** a spacecraft (purple dot) is located near a scattering boundary. In the plane perpendicular to the magnetic field, the particle detector needs to collect electrons from all directions to get a full perpendicular PSD (PSD_⊥_). However, the measured PSD_⊥_ is not gyrotropic because electrons outside the boundary can be scattered away by the boundary. Two critical looking directions (*ϕ*_1_ and *ϕ*_2_), corresponding to the gyro-orbits tangential to the boundary (two cyan circles), can be recognized by sharp PSD declines. The two critical directions as well as the particle gyro-radius can be used to calculate the boundary orientation (*β*_*i*_) and distance (*R*_*i*_) to the spacecraft. Panel **b** shows an example of boundary sounding. Using PSD_⊥_ data for a certain energy channel, the sounding technique generates a data point (red) at each moment, leading to a series of orientations (*β*_*i*−3_*,…, β*_*i* + 3_) and distances (*R*_*i*−3_*,…, R*_*i*+3_) throughout the whole traversal. The corresponding data points (red crosses) are obtained based on the known spacecraft location. The boundary geometry can be obtained by connecting these points

The schematic of the sounding technique is shown in Fig. 7. In panel a, a spacecraft is located inside an electron trapping region and the distance to the boundary is less than twice the gyro-radius. For some look direction in the magnetic perpendicular plane, if the corresponding particle gyro-orbit intersects the boundary, the particle flux received by the detector in this direction will be significantly reduced due to the scattering at the boundary. Thus, the measured perpendicular PSD (PSD_⊥_) will be non-gyrotropic, and two critical look directions can be recognized by sharp reductions, which correspond to two special gyro-orbits that are just tangential to the boundary. The relationship between the boundary orientation (*β*) and distance (*R*) to the spacecraft, and the two critical look directions (written as *ϕ*_1_ and *ϕ*_2_), is given as^40^:

5 $$\beta = \frac{{\phi _1 + \phi _2}}{2} + \frac{{\mathrm{\pi }}}{2},$$

6 $$R = \rho - \rho \cdot \cos \left( {\frac{{\phi _2 - \phi _1}}{2}} \right).$$

Practically, the sounding technique can give a series of orientations (*β*_*i*−3_*,…, β*_*i*+3_) and distances (*R*_*i*−3_*,…, R*_*i*+3_) from observations at different moments, thus a sequence of boundary points is obtained, as illustrated in panel b of Fig. 7. Note that the sounding technique can be applied to PSDs of different energy channels. In reality, combined observations from multi-spacecraft and multi-energy channels can significantly increase the number of data points from the sounding technique.

The uncertainty of the sounding results is previously given considering the uncertainty of the energy and the look direction^40^. If we take the spacecraft motion into account, the uncertainty is given by:

7 $$\Delta R = \sqrt {\left( {2R\rho - R^2} \right)\Delta \phi ^2 + \left( {\frac{R}{\rho }\Delta \rho } \right)^2 + \left( {V\Delta t} \right)^2},$$

where *R* is the distance obtained by the sounding technique, *ρ* is the particle gyro-radius, ∆*ρ* is the uncertainty of *ρ*, ∆*ϕ* is the uncertainty of the direction, *V* is the spacecraft speed relative to the plasma rest frame (approximated by *V*_p_), and ∆*t* is the time resolution. A supremum of ∆*R* is evaluated to be 2.2 km in the ESMC event discussed in this paper.

### Correction due to a curved boundary

**Fig. 8:**
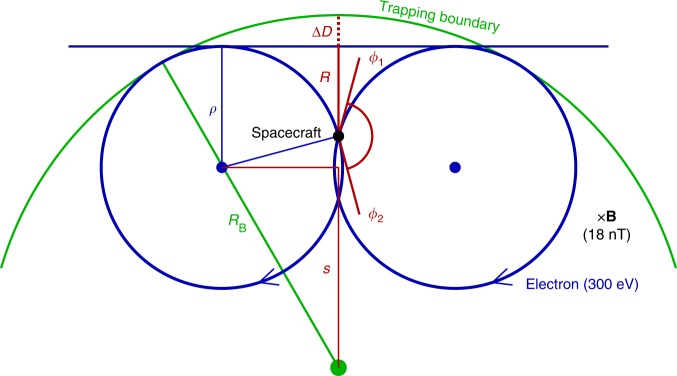
The underestimation of the technique if the actual boundary curves outward. ∆*D* is the underestimation, *ρ* is the particle gyro-radius, *R*_B_ is the boundary curvature radius, *R* is the sounding distance, and *ϕ*_1_ and *ϕ*_2_ are critical angles derived from the sounding technique

Another factor that may affect the accuracy of the sounding result is the boundary curvature. Generally, for a trapping boundary of unknown shape, it is a natural to assume a planar boundary. For an actual boundary that is not planar, the sounding technique will overestimate (underestimate) the distance, if the boundary curves inward (outward). For a magnetic cavity event, the sounding technique will underestimate the distance, as illustrated by Fig. 8. This systematic underestimation can be computed from particle gyro-radius *ρ*, boundary curvature radius *R*_B_, and the sounding distance *R*, as given by

8 $${\mathrm{\Delta }}D = R_{\mathrm{B}} - \rho - s = R_{\mathrm{B}} - \rho - \sqrt {\left( {R_{\mathrm{B}} - \rho } \right)^2 - \left( {\rho ^2 - \left( {\rho - R} \right)^2} \right)},$$

where the parameter *s* is schematically shown in Fig. 8.

An average of underestimations ⟨Δ*D*⟩ for all sounding results could be a correction of the scale size. However, the change of the scale size *R*_B_ will affect the underestimation ∆*D* itself. Thus, an implicit correction method is proposed:

9 $${\mathrm{\Delta }}D = \left( {R_{\mathrm{B}} + {\mathrm{\Delta }}L} \right) - \rho - \sqrt {\left( {R_{\mathrm{B}} + {\mathrm{\Delta }}L - \rho } \right)^2 - \left( {\rho ^2 - \left( {\rho - R} \right)^2} \right)},$$

10 $${\mathrm{\Delta }}L = \langle{\mathrm{\Delta }}D\rangle.$$

A careful calculation of ∆*L* shows that ∆*L* ~0.47 km, which is smaller than the estimated supremum of uncertainty ∆*R* (2.2 km). In the main text, Fig. 3 shows the data points with the correction method already been applied.

## Supplementary information

Supplementary Information

## Data Availability

The datasets analyzed during the current study are publicly available from the MMS Science Data Center (https://lasp.colorado.edu/mms/sdc/public/), including burst mode magnetic fields from the Fluxgate Magnetometers (FGM) and the Search Coil Magnetometers (SCM), and burst mode proton and electron distributions from the Fast Plasma Investigation (FPI).
