# Supplementary material for: MMS observations of electron scale magnetic cavity embedded in proton scale magnetic cavity
Source: Nat Commun. 2019 Mar 4;10:1040. doi: 10.1038/s41467-019-08971-y (PMC6399300; doi:10.1038/s41467-019-08971-y)
Supplement: Supplementary file 1 — Supplementary Information [file 41467_2019_8971_MOESM1_ESM.pdf]

Supplementary Information

MMS observations of electron scale magnetic cavity embedded in proton scale magnetic cavity

Han et al.

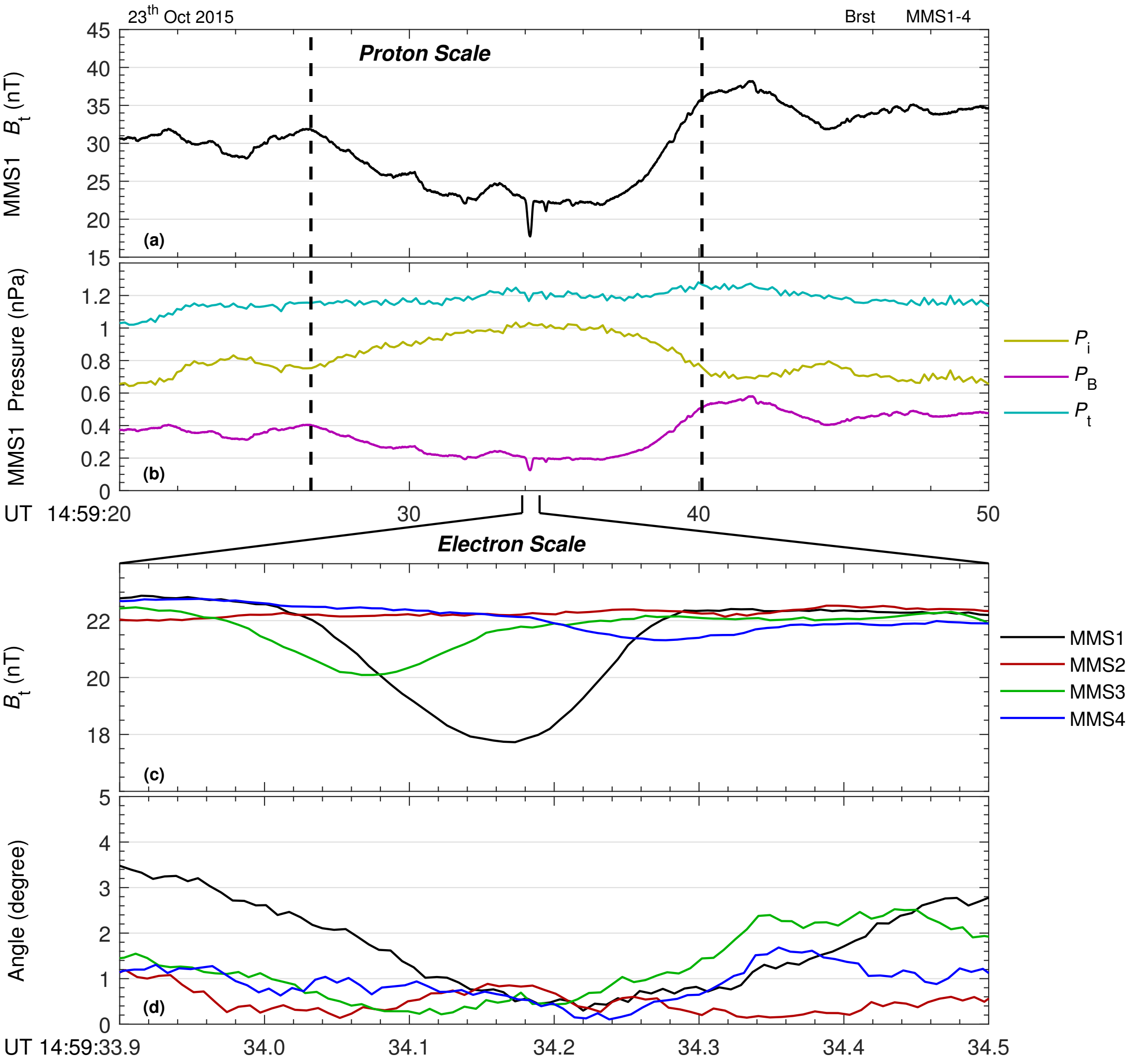

Supplementary Fig. 1 Pressure balance at proton scale and magnetic field directions at electron scale. Panels (a) and (b) show MMS1 observations in 30 s time interval from 14:59:20UT to 14:59:50UT. Panel (a) shows the magnetic field strength (B_t). In panel (b), three lines denote proton thermal pressure (P_i), the magnetic pressure (P_B), and total pressure (P_t), respectively. Two vertical dashed lines mark a depression region of B_t. Panels (c) and (d) present electron scale observations of B_t and magnetic field directions from MMS1-4. The field directions are indicated by angles relative to the average magnetic field, which is used as the Z-axis of a newly defined plasma rest frame, detailed in the main text.

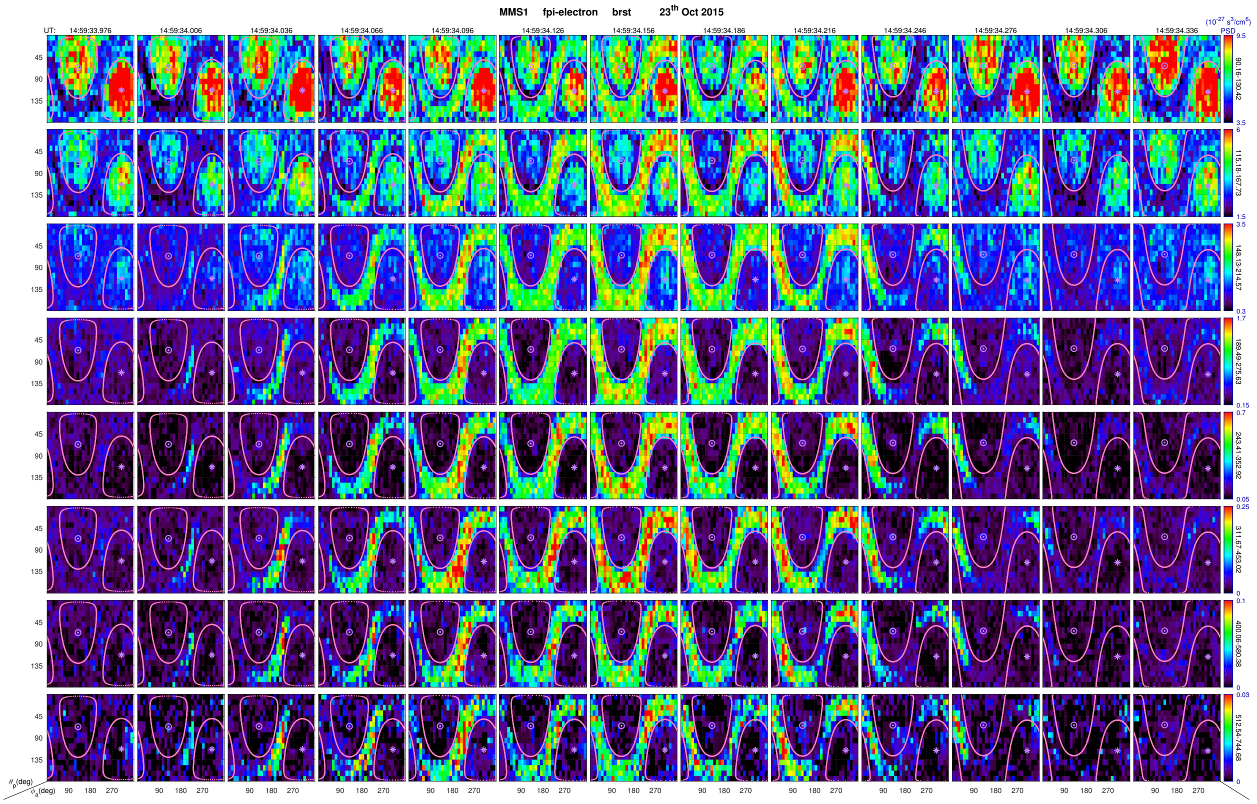

Supplementary Fig. 2 Sky-maps of electron phase space density (PSD) from MMS1 for multiple energy channels. Panels in the same row shares a common energy channel and a common color bar, but are measured at different times. For a specific panel in this huge figure, the corresponding energy channel is labeled on the far right, and the measuring time is labeled on the top of the whole column. For each panel the horizontal and vertical axes are azimuthal (ϕ_a, degrees) and polar angles (θ_p, degrees) in the DBCS coordinates, respectively. Magenta lines on each panel represent the local loss cone, detailed in the main manuscript. The asterisk and the circle mark directions parallel and anti-parallel to the magnetic field, respectively.

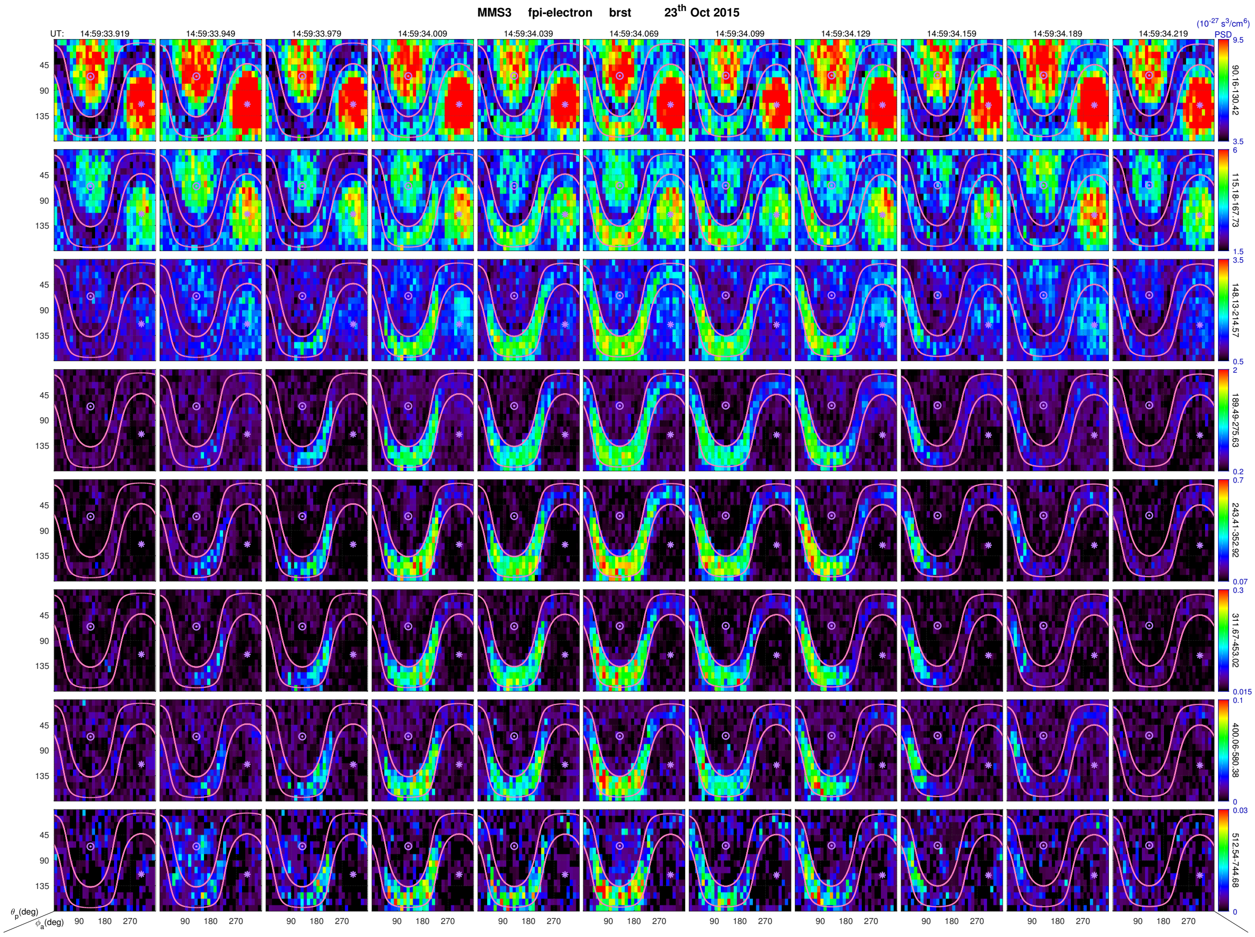

Supplementary Fig. 3 Sky-maps of electron phase space density (PSD) from MMS3 for multiple energy channels. The same as Supplementary Fig. 1 but for MMS3.

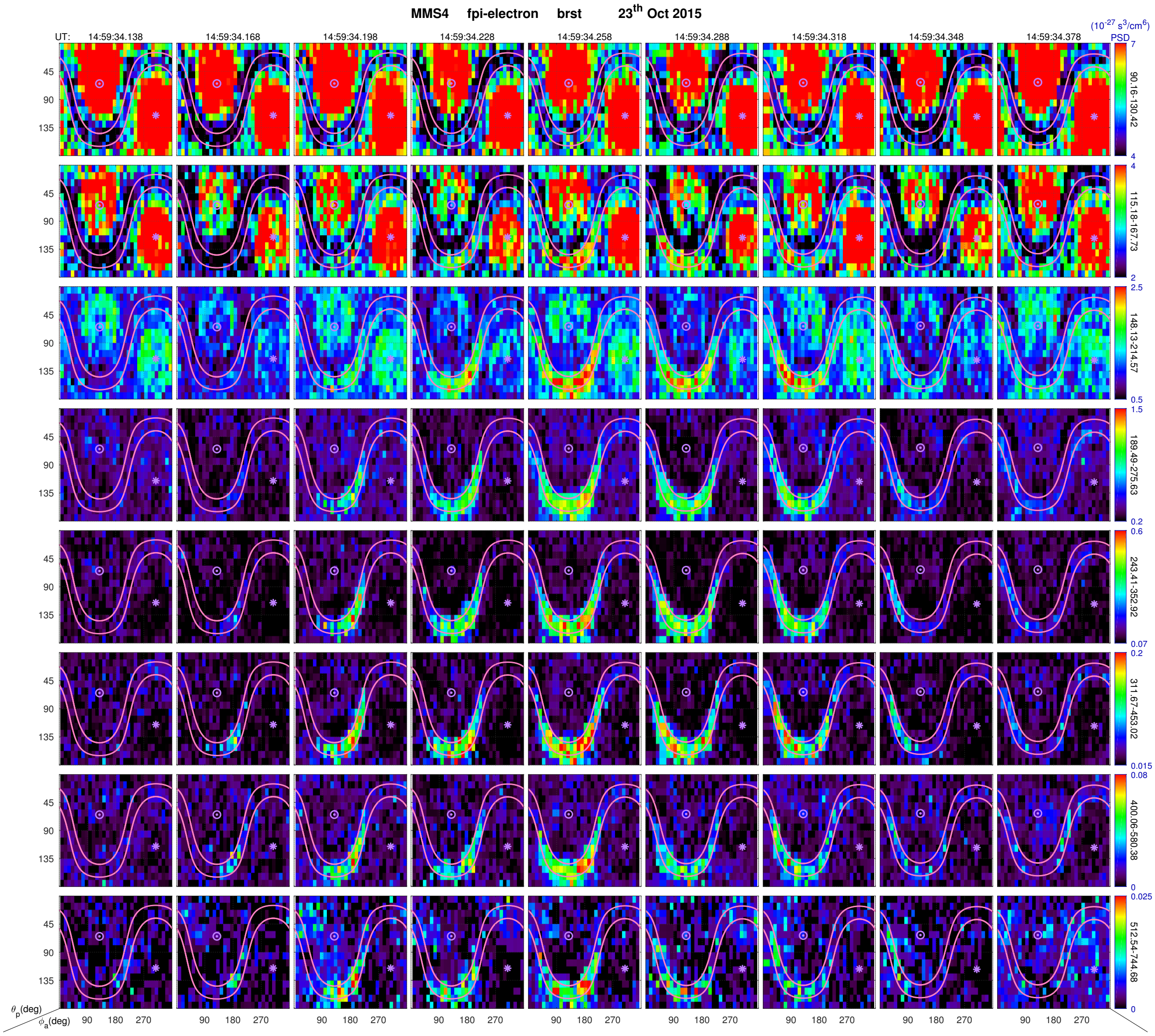

Supplementary Fig. 4 Sky-maps of electron phase space density (PSD) from MMS4 for multiple energy channels. The same as Supplementary Fig. 1 but for MMS4.
